# Engineering Botulinum Toxins to Improve and Expand Targeting and SNARE Cleavage Activity

**DOI:** 10.3390/toxins10070278

**Published:** 2018-07-04

**Authors:** Elena Fonfria, Mark Elliott, Matthew Beard, John A. Chaddock, Johannes Krupp

**Affiliations:** 1 Ipsen Bioinnovation, 102 Park Drive, Milton Park, Abingdon OX14 4RY, UK; mark.elliott@ipsen.com (M.E.); matthew.beard@ipsen.com (M.B.); john.chaddock@ipsen.com (J.A.C.); 2 Ipsen Innovation, 5 Avenue du Canada, 91940 Les Ulis, France; johannes.krupp@ipsen.com

**Keywords:** recombinant, target secretion inhibitors, rational design, receptors, enzyme

## Abstract

Botulinum neurotoxins (BoNTs) are highly successful protein therapeutics. Over 40 naturally occurring BoNTs have been described thus far and, of those, only 2 are commercially available for clinical use. Different members of the BoNT family present different biological properties but share a similar multi-domain structure at the molecular level. In nature, BoNTs are encoded by DNA in producing clostridial bacteria and, as such, are amenable to recombinant production through insertion of the coding DNA into other bacterial species. This, in turn, creates possibilities for protein engineering. Here, we review the production of BoNTs by the natural host and also recombinant production approaches utilised in the field. Applications of recombinant BoNT-production include the generation of BoNT-derived domain fragments, the creation of novel BoNTs with improved performance and enhanced therapeutic potential, as well as the advancement of BoNT vaccines. In this article, we discuss site directed mutagenesis, used to affect the biological properties of BoNTs, including approaches to alter their binding to neurons and to alter the specificity and kinetics of substrate cleavage. We also discuss the target secretion inhibitor (TSI) platform, in which the neuronal binding domain of BoNTs is substituted with an alternative cellular ligand to re-target the toxins to non-neuronal systems. Understanding and harnessing the potential of the biological diversity of natural BoNTs, together with the ability to engineer novel mutations and further changes to the protein structure, will provide the basis for increasing the scope of future BoNT-based therapeutics.

## 1. Botulinum Toxin Background

Botulinum neurotoxin (BoNT) has been used as a versatile and multipurpose therapeutic for the last 30 years [1,2,3]. In 2015 the global BoNT market was valued at $3.4 billion US dollars and is expected to grow to $7.3 billion by 2025 [4]. The current preparations of BoNT approved for clinical use in the US and Europe correspond to two different active molecules—BoNT type A (BoNT/A) and BoNT type B (BoNT/B): abobotulinumtoxinA (Dysport^®^, Ipsen, Paris, France), incobotulinumtoxinA (Xeomin^®^, Merz, Frankfurt, Germany), onabotulinumtoxinA (Botox^®^, Allergan, Dublin, Ireland)—and rimabotulinumtoxinB (Myobloc^®^, Solstice Neurosciences, San Francisco, CA, USA). A further six types of BoNT exist in nature, named BoNT type C to G. Recently, new BoNT types have been described, such as type FA (previously classified as H), and several of them are being explored as new therapeutic agents [5,6,7].

In nature, BoNTs are produced by several bacteria of the genus Clostridia [8], and more than 40 different BoNT protein sequences have been described to date [9]. At the molecular level, all BoNTs broadly share the same structure [10,11], and the structure-activity relationship of the BoNT domains is credited for the selectivity and extreme potency of the molecules [12]. BoNTs are widely regarded as the most potent toxin known to man, with an estimated human lethal dose of 1.3–13 ng/kg intravenously, intramuscularly or when inhaled [13].

BoNT selectively target the neuromuscular junction and, in particular, exerts its effect at the level of nerve innervation of the muscle. It is the muscle paralysis in intoxicated individuals that causes harm in the form of botulism, and in the therapeutic setting brings relief to movement disorders such as dystonia and spasticity [14].

Beyond muscle paralysis, BoNT therapeutics are also used for glandular hypersecretion, for example in hyperhidrosis and sialorrhea [15]. Furthermore, BoNTs are reported to also affect inhibitory and excitatory neurons, and also sensory neurons [16]. Recently, non-neuronal effects of BoNTs have also been described in human skin and other tissues, which could further expand the future utility of BoNTs [17]. 

Our increasing understanding of the molecular structure of BoNTs and the relationships between each domain and their function is opening up both the ability to produce natural BoNTs in a recombinant manner [18] and also the ability to modify BoNTs using protein engineering [19].

## 2. Recombinant Production and Manufacture

Research using BoNT, both native and recombinant, needs to comply with relevant national legislation. For example, in the US, BoNT is classified as a Category A Tier 1 agent by the Centres of Disease Control and Prevention (https://emergency.cdc.gov/), and there is a requirement for facilities where research is conducted with select agents and toxins to register with the Federal Select Agent Program (FSAP), which regulates the possession, use and transfer of biological select agents and toxins (for further information please see https://www.selectagents.gov/index.html). In the UK, BoNTs are listed in Schedule 5 of the Anti-terrorism, Crime and Security Act 2001, and it is a legal requirement for all facilities in the UK who hold and/or use Schedule 5 substances to be registered with the Home Office and have stringent materials and personnel security measures in place (for further information please see https://www.gov.uk/government/organisations/home-office).

At the time of writing this article, all commercially available BoNT preparations are sourced from native Clostridium botulinum bacterial hosts. For review see Pickett, 2014 [20], and specifically Table 1 within the review, for main characteristics of the major BoNT products available in Europe and the US. 

When BoNT is expressed and harvested from Clostridia sp., it is as a complex of the active pharmaceutical ingredient 150 kDa BoNT and a mixture of neurotoxin-associated proteins (NAPS) that include non-toxic non-haemagglutinin (NTNH) and haemagglutinin (HA) proteins [21]. For a review of the proteins associated with BoNT, their characteristics, and potential purpose see Pirazzini et al. [22]. Two of the commercially available BoNT/A products, abobotulinumtoxinA and onabotulinumtoxinA, are purified toxin complexes, whereas the third commercially available BoNT/A product, incobotulinumtoxinA, contains the purified BoNT/A without NAPS.

Although the high potency of BoNT products provides assay characterisation challenges, it does mean that manufacturing of material within the research phase, or even during pre-clinical and clinical development, requires relatively low volume fermentation equipment and small scale downstream processing. With such small-scale protein needs, a wide variety of opportunities exist for expression and harvesting of BoNTs, beyond extraction from Clostridia; many of which are based on recombinant DNA technology. Acknowledging the physical and chemical constraints that exist within protein biochemistry, in that protein structure/function can be significantly adversely affected by an attempt to modify even just one amino acid within a protein sequence, the possibility to manipulate protein structure by modification of the encoding DNA is an attractive and versatile approach to manipulating the properties of proteins [23].

When considering the success of recombinant approaches, the choice of expression host has a significant bearing on the expressed protein and so a wide variety of bacterial, yeast, mammalian and in vitro systems are available to be used for expression of proteins from a variety of origins. Commonly, and certainly the case within recombinant BoNT (rBoNT) expression approaches, the gram-negative bacterium Escherichia coli (*E. coli*) is an expression host of great simplicity and utility and will be the subject of most of the later discussion. Beyond *E. coli*, the use of yeast, such as Pichia [24] and Lactobacillus [25] for expression of BoNT domains for potential vaccine use have been successful. Closer to ‘home’, nonsporulating and nontoxigenic *C. botulinum* expression host strains have been developed [26] that allow BoNTs to be expressed within a clostridial host. Furthermore, the understanding of Clostridia sp. genetics is facilitating the utilisation of the native host for exploration of BoNT biology [27].

Through the application of recombinant DNA and expression techniques, there is an emerging body of literature on the usefulness of such an approach in the creation of multi-domain rBoNTs, recombinant domains of BoNTs, rBoNTs with modified properties (for example with regard to soluble N-ethylmaleimide-sensitive factor attachment protein receptor (SNARE) protein cleavage characteristics or cell binding properties), rBoNTs with beneficial biochemical properties, rBoNTs and domains optimised for high level expression, rBoNT tool molecules for assay development, and additional uses. Such techniques have also been applied to the structurally similar tetanus toxin [28]. 

The ability to modify the primary sequence of the expressed BoNT or BoNT fragments has also facilitated new approaches to coupling protein domains together (as exemplified by the stapling approach described in [29], the sortase coupling approach as described by Zhang et al. [30] or the conjugate approach described by Nugent et al. [31]) to supplement the more traditional recombinant approach of synthesising a single polypeptide chain incorporating multiple protein domains from a single engineered DNA coding sequence. Such techniques have been utilised to develop BoNT derivatives with properties that differ from the native sequence toxins. For example, the protein stapling approach has been used to derive BiTox, whereby the LH_N_ (BoNT fragment comprising LC and the translocation domain H_N_) and the binding domain (H_C_) of BoNT/A are ‘stapled’ together via a SNARE complex formed by complimentary fragments of SNAP-25, VAMP-2 and syntaxin. BiTox is reported to be non-paralytic, but possesses anti-nociceptive properties [32]. In an earlier study of BiTox, up to a 200 ng/kg dose i.p. in mice showed no observable signs of muscle weakness within 4 days, whereas BoNT/A was lethal even at 2 ng/kg within 24 h [29]. In that study, the authors hypothesise that the resulting architecture of the new molecule, with the incorporation of the “staple” peptides, would preclude the efficient internalization of the toxin in the motor neurons due to steric hindrances. Regarding pain mechanisms, it was shown in the later study [32] that a single intraplantar injection of 200 ng/rat of BiTox was nonparalysing but effective in attenuating both A-nociceptor-mediated secondary mechanical hyperalgesia and neuropathic pain in rats. Furthermore, BiTox did not reduce C-mediated nociception but inhibited plasma extravasation and inflammatory oedema and reduced keratynocite proliferation local to the site of toxin injection.

The versatility of recombinant techniques for the expression and preparation of BoNTs and BoNT domains have led to exploration of BoNTs as intracellular delivery vehicles for protein cargo [33,34] and allowed the preparation of hybrid BoNTs that exhibit preferred characteristics by utilising properties of, for example, two different serotypes of BoNT [35,36,37]. In terms of providing engineering opportunities, recombinant approaches have the potential to facilitate site specific labelling of the protein which could have application in inter-/intra-cellular trafficking studies, elimination of unwanted characteristics (such as chemical liabilities), inclusion of specific protein purification tags to enable swift and effective isolation from crude media, and importantly the generation of tool molecules to explore BoNT mechanism of action. Finally, expression of BoNTs in non-clostridial hosts can overcome many of the disadvantages outlined earlier, and brings the opportunity of developing a suite of therapeutics with enhanced properties. Such enhanced properties can be advantageous for the manufacturing process; for example, by providing a consistent, scalable manufacturing approach free of concerns of sporulation and using *E. coli* hosts that are genetically modified for lack of survival in the non-lab environment. Careful consideration must be given to the choice of strain and plasmid, and common problems encountered during recombinant protein expression in *E. coli* include no or low expression of the protein of interest (possibly due to the protein being toxic before or after induction, or codon bias), formation of inclusion bodies (possibly due to incorrect disulphide bond formation, incorrect folding, low solubility of the protein, or the need of an essential post-translational modification), and protein inactivity (possibly due to incomplete folding or mutations in the cDNA) [38].

As with any new product, recombinant BoNTs need to be thoroughly researched and analysed through a plethora of pre-clinical tests to understand, as far as possible, their pharmacodynamics and expected safety profile, prior to testing in humans. Considering the regulatory aspects of rBoNTs further, Weisemann et al. [18] recently described the preparation of a suite of rBoNTs for use as test standards. By utilising recombinant techniques to express well characterised, active di-chain toxins linked by a disulphide bridge with highly accurate protein concentration estimations, the authors note the usefulness of this material in providing standards as spiking material in a worldwide BoNT proficiency test organized by the EQuATox (Establishment of Quality Assurances for the Detection of Biological Toxins of Potential Bioterrorism Risk) consortium. 

As noted earlier, all current BoNT FDA-approved products are derived from natural Clostridia sources. However, it is now clear that the ability to modify BoNT domains affords the drug development researcher the opportunity to create novel BoNTs that have improved properties. An important, but often overlooked, inherency of achieving the stage gate of transitioning into the clinic with a complex biologic like BoNT or BoNT-derived multidomain proteins, is the demonstration to regulatory agencies (e.g., EMA or FDA) that the material can be manufactured to a quality suitable for use in humans. By entering the clinical trial phase (rBoNT/E) or being clinical development ready [39], manufacturers have demonstrated the suitability of recombinant approaches to the preparation of BoNT and biologics based on BoNT fragments.

In addition to the therapeutic opportunities afforded by the application of recombinant techniques to BoNT engineering and manufacture, a second vital application opportunity is in the field of vaccine production [40,41]. For many years, researchers have explored the use of domains/fragments of BoNT as candidates for the preparation of new vaccines to BoNTs. With the number of natural BoNT variants expanding all the time, and the recent discovery of BoNT-like genes in non-clostridial hosts [42], the need for safe, effective vaccines and/or immunotherapy approaches is more pressing than ever. Recombinant expression techniques allow multiple domains and BoNT fragments to be explored for vaccine effectiveness [43,44].

When utilising non-native expression techniques, as in the case of expression rBoNT/s in a non-clostridial host, one aim is to produce a recombinant protein that is equivalent to its native sourced counterpart. LC, and H_C_ BoNT domains are robust polypeptides that have been successfully expressed in non-clostridial hosts; by contrast the H_N_ translocation domain has proved challenging to obtain in isolation, although this has been achieved through protein refolding techniques [45]. Although the natural BoNT complex proteins are not present in the rBoNT material, rBoNTs have been shown to be robust, stable, and efficacious proteins. Furthermore, recombinant techniques have enabled characterization of the BoNT complex: the work of Lee et al. [46] reporting the reconstitution of the 14 subunit complex from recombinantly produced components and the impact of complex formation on oral toxicity.

## 3. BoNT Targeting

BoNTs interact with their target cell surface in a dual step binding mode, a hall-mark feature that enables their high specificity in targeting cholinergic motoneurons [47,48]. The dual binding mode for most BoNT serotypes consists of binding to polysialylated gangliosides coupled with binding to specific membrane proteins of the presynaptic terminal. BoNT/A, /E and /F bind to synaptic vesicle proteins isoform 2A-C (SV2A-C), whereas BoNT/B, /G, and the mosaic toxin /DC bind synaptotagmin 1 and 2 (Syt 1, 2) [8,22,48,49,50,51,52]. BoNT/C binds to two gangliosides to gain cellular entry [53], whereas the receptors of BoNT/D and tetanus toxin remains to be clarified. Tetanus toxin may explore SV2 and/or nidogens as receptors [54]. BoNT/D may use SV2 as a receptor [55]. The cell binding modalities of a recently described BoNT/X have yet to be explored [30].

Kinetic studies indicate that binding affinities of BoNTs to gangliosides may be in the high nM range [56,57]. However, a recent study has reported binding affinities to gangliosides that are significantly smaller than that [58]. In contrast, most studies agree that binding affinities of BoNTs to their protein receptor is typically in the low µM/high nM range [59,60,61]. Steady-state binding constants for the dual binding are in the high pM range [62,63,64,65]. When comparing the binding and unbinding rates of BoNTs bound only to the protein receptor with those of the dually bound BoNTs, it appears that the higher affinity of the doubly bound BoNT may primarily result from significantly reduced unbinding rates [65]. This would indeed increase the likelihood of endocytosis completion prior to unbinding. Recently, a tri-partite recognition model has been proposed, with the third element of cellular recognition being a hydrophobic loop in the H_C_ domain that would bind lipids in the plasma membrane [66]. Binding kinetics and thermodynamic analysis of wild type H_C_ loop and of H_C_ loop mutants revealed a crucial role in binding affinity (e.g., in the range of nM/ high nM for affinities of the wild type H_C_/B, H_C_/DC and H_C_/G, severely reduced or obliterated when the loop is mutated), and the authors also proposed a functional role during the translocation step.

The carboxyl-terminal subdomain of H_C_ (H_CC_)-domains of BoNT/A, /B, /E, /F, /G, as well as the tetanus toxin possess a conserved ganglioside-binding motif of the general amino acid structure H_BoNT/A,/B,/F,TeNT_/K_BoNT/E,G_…SXWY…G [57,67,68,69,70]. Whereas the SXWY motif in its strict sense is not found in BoNT/C and /D, their ganglioside binding site is analogous [71,72,73,74,75]. The details of how these toxins interact with gangliosides remains to be fully elucidated. The mosaic toxin BoNT/DC has recently been shown to have a unique ganglioside interaction mechanism, recognizing only the sialic acid moiety of gangliosides [76].

Recent studies have also provided insight into the way BoNTs interact with their protein receptors. For example, the molecular basis of the BoNT/B–Syt interaction has been determined by X-ray crystallography [77,78,79], showing that Syt interacts in an induced α-helical configuration via side-chain interactions with the BoNT H_CC_ domain. A strong affinity of BoNT/B for the rodent Syt 2 receptor is based mainly on interactions across two hydrophobic pockets of the binding cleft. Of note, the phenylalanine 54 in the rodent Syt 2 sequence is replaced by a leucine in the human sequence. The absence of the benzyl ring in the side-chain of the human residue dramatically decreases the affinity to BoNT/B [80,81], likely explaining the high doses needed in the clinic when using the available BoNT/B product [82].

Compared to the BoNT/B–Syt interaction described above, the binding of BoNT/A, /E and /F to SV2 proteins is quite different from a molecular point of view, as SV2 proteins present a prearranged, β-sheet configuration to interact in a backbone to backbone interaction with BoNTs at the H_CC_ and H_CN_ interface. Furthermore, it has been shown that glycosylation of a conserved asparagine in SV2 proteins (N559) has strong effects on unbinding constants, and thus is crucial for high affinity interaction with BoNTs [59,60]. There is evidence that BoNT/A and /E do not interact with all three SV2 isoforms to the same degree and with the same affinity. For example, BoNT/E preferentially interacts with glycosylated SV2A and SV2B, but not with SV2C [83]. In contrast, although BoNT/A can interact with all three SV2 isoforms, it prefers SV2C [59].

**Figure 1:**
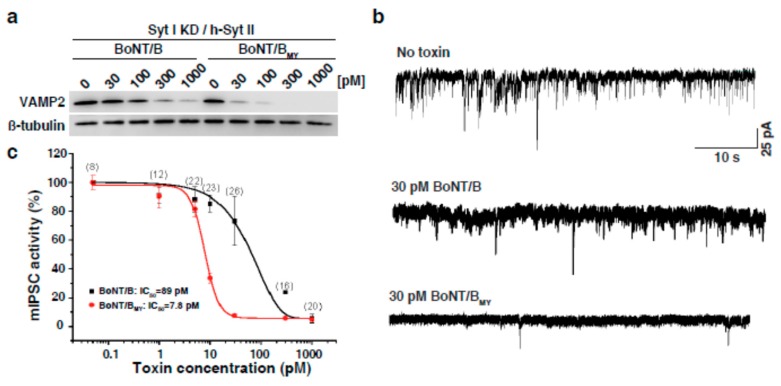
(**a**) BoNT/B1 protein modified to have high affinity to human SYT2 (BoNT/BMY) shows enhanced efficacy in neurons that express h-Syt II [61]. Briefly, humanized neurons were created by knockdown of endogenous Syt I and by expressing full-length h-Syt II in cultured rat cortical neurons via lentiviral transduction. Panel A shows the amount of VAMP2 cleaved by BoNT/B or the modified BoNT/B following 24 h of exposure. β-tubulin expression was used as loading control. Panel (**b**) and (**c**) shows humanized neurons exposed to BoNT/B or modified BoNT/B for 24 h. The mIPSC was recorded by a whole-cell patch-clamp approach. Panel (**b**) shows representative mIPSC recordings at 30 pM toxins, whereas panel (**c**) shows the mIPSC activities versus toxin concentrations, normalized to neurons that were not exposed to toxins. For further details please see [61].

Given the rapidly increasing knowledge on the molecular interactions between BoNTs and their ganglioside and protein receptors, the possibility to rationally alter such interactions through engineering has become a real option for future development of new BoNT therapeutics. In this endeavour, our knowledge about the biology of the receptors must guide the engineering strategies. For example, the above described species differences in Syt 2 may allow to develop BoNT/B toxins that overcome the drawbacks associated with the present BoNT/B product. Likewise, our knowledge about the expression pattern of the different BoNT receptors provides a rich source for engineering strategies. For example, while Syt 2 is the predominant isoform at neuromuscular junctions of striated muscle [84,85,86], Syt 1 is the predominant isoform in autonomic and sensory neurons [87]. Furthermore, there is a species difference in the Syt 2 protein, which results in BoNT/B displaying a high affinity to the rodent Syt 2 isoform but a rather low affinity to the human Syt 2 homologue [80,81]. Previous insights into the molecular basis of the BoNT/B–Syt interaction obtained by X-ray crystallography [77,78,79], combined with a bacterial adenylate cyclase two-hybrid saturation mutagenesis screen, allowed identification of a series of point mutations that can be introduced into the receptor-binding domain of BoNT/B to enhance its binding to human Syt 2 [61]. One of the identified double mutations, E1191M/S1199Y, was incorporated into a full-length active BoNT/B1 toxin, which resulted in increased functional efficacy in a humanized cell model [61], Figure 1. Given the variety of additional mutations that were identified in the Tao et al. study, a rich palette of possible mutations for further exploration has been provided.

While all three SV2 proteins are expressed in neuronal cells, the predominantly expressed SV2 protein can vary between brain regions as well as the main neurotransmitter [88]. Furthermore, there is also evidence that specific SV2 isoforms are up- or down-regulated in specific clinical conditions [88,89]. Like SV2 proteins, brain gangliosides also are vastly regulated during development and disease states, and their regulation has been implicated in central nervous system diseases like Alzheimer’s Disease, Parkinson’s Disease or Huntington’s disease [90,91], but also in neuromuscular disease like the Gullain Barré Syndromes [92]. Given the multitude of options for targeted modifications, it is unsurprising that efforts are under way to alter the receptor–toxin interaction properties with the aim of increased and more specific interaction. In the following section, we shall review three such efforts.

While the H…SXWY…G ganglioside binding motif can be directly modified to down-regulate ganglioside binding, for example through mutation of W1266 (BoNT/A) or W1262 (BoNT/B) by leucine [68], it has also been shown that residues sequentially outside the motif can modify the ganglioside interaction. For example, a residue prior to the SXWF motif, Y1117, has been shown to have strong effects on ganglioside binding and activity in a hemidiaphragm assay when mutated in BoNT/A [93]. Additional residues with strong effects on activity in or around the SXWF motif, including F1252, H1253, Q1270, L1278, or G1279, have been identified [93]. Of note, substitutions of these residues can be found that increase ganglioside binding affinity in rat brain synaptosomal preparation as well as activity in ex vivo hemidiaphragm assays [93].

Another example of ganglioside binding modification is the demonstration that it is possible to change the ganglioside selectivity of the BoNT/F serotype [57]. Comparing the ganglioside binding region of BoNT/F with the ganglioside binding region of other BoNT serotypes allowed the authors to identify H1241 in BoNT/F as a possible determinant of ganglioside selectivity. Indeed, exchange of the histidine at this position to the corresponding lysine residue of BoNT/E resulted in increased affinity for GD1a of the mutated BoNT/F and, most noteworthy, the ability of this mutated BoNT/F to bind GM1a [57].

## 4. BoNT Re-Targeting

The therapeutic usefulness of native sequence, or modified recombinant BoNTs is broadly underpinned by the natural targeting of the toxin to the neuromuscular junction. The ability of BoNT LC to efficiently and selectively cleave SNARE proteins, combined with their long duration of effect, make them an attractive payload for delivery into cells that are not natural BoNT targets, allowing their therapeutic use beyond neurons. Altering the cell selectivity of BoNT allows delivery of the light chain to different cell types, thereby unlocking the enormous potential of this protein beyond the neuromuscular junction.

Efforts to retarget the toxin have been greatly facilitated by the exquisite modular structure-function relationship of the BoNT protein. A fragment of BoNT/A composed of the LC and H_N_ domains (LH_N_) was discovered following treatment of the full-length toxin by trypsin [94]. Since it lacks the H_C_ domain, LH_N_ is unable to bind to neuronal cells with high efficiency, which reduces its toxicity considerably compared to the full-length toxin. However, LH_N_ retains a fully active catalytic domain and translocation domain, allowing it to form pores in membranes under acidic conditions [95]. Such a combination means that once internalised into a cell through the addition of a suitable binding domain, the translocation of the LC into the cytosol via the membrane pore formed by H_N_ can occur. The crystal structure of LH_N_/A revealed that the LC and H_N_ domains retained their structural relationship demonstrated in the crystal structure of BoNT/A, despite the absence of the H_C_ domain [96]. These properties have made the LH_N_ fragment the most common platform on which retargeted toxins have been based for delivery of the LC into cell types beyond the reach of BoNT.

The potential of the LH_N_ fragment for delivery of LC endopeptidase into cells was first demonstrated using chemical conjugation of a targeting entity. Early work on this type of approach (frequently referred to as Targeted Secretion Inhibitors, TSI) involved the use of a LH_N_/A preparation purified from full-length BoNT/A. Using this strategy of chemical coupling, the lectin wheat germ agglutinin and nerve growth factor (NGF) were chemically linked to LH_N_/A, with the resulting conjugates able to inhibit the release of noradrenaline from PC12 cells [97]. In both cases, the conjugated molecules were significantly more effective than the un-liganded LH_N_/A alone in cleaving SNAP-25 and inhibiting secretion from the target cells, thus demonstrating that the majority of internalisation was occurring via the receptor to which the molecule was targeted. These studies showed that the endopeptidase could be successfully delivered into a range of different cell types, both neuronal and non-neuronal.

Later efforts have been greatly improved by the recombinant expression and purification of the LH_N_ fragment from *E. coli* [98]. The recombinant approach has enabled production of fusion proteins combining LH_N_ with a targeting entity—for example a peptide ligand. Addition of an exogenous protease site within the activation loop between LC and H_N_ allows production of active di-chain proteins. Although initial studies were conducted using LH_N_/A as the basis for the re-targeted molecules, alteration of the serotype from which the LH_N_ is derived affords an extension to the platform for the cleavage of an extended array of SNARE proteins. This is particularly important when considering targeting of non-neuronal cell types, which rarely express the substrate of LC/A, SNAP-25. For example, LH_N_/C coupled to epidermal growth factor (LH_N_/C-EGF) was shown to inhibit the release of mucin from pulmonary epithelial A549 cells, demonstrating the potential of such molecules for the treatment of disorders such as asthma and chronic obstructive pulmonary disease (COPD) [99].

One therapeutic area where re-targeted BoNTs may be particularly effective is the treatment of chronic pain conditions, through the changing of target specificity from motor neurons to peripheral sensory afferent neurons. An early demonstration of the potential for retargeting BoNTs to nociceptive neurons was provided by conjugating LH_N_/A with Erythrina cristagalli (ECL), a lectin able to bind to galactose-containing carbohydrates present on the surface of mammalian cells. Further work by the same group using the recombinant TSI platform, combined different combinations of LH_N_ and peptide ligands to target the molecules to G-protein coupled receptors (GPCRs) known to be present on nociceptive neurons. These efforts produced several molecules that demonstrated analgesic properties in vivo with no adverse effects and no activity at the NMJ at therapeutically relevant doses. One of these TSIs, (named Senrebotase) targeted to the nociceptin receptor (OPRL1), was selected for clinical development by Allergan. (see https://clinicaltrials.gov/, NCT01678924 and NCT01157377). 

Ma et al. targeted the P2X purinoceptor 3 (P2X3), using a single-chain variable fragment (scFv) antibody (~25 kDa) generated against the extracellular domain of the receptor (designated MH7C) [100]. A recombinant protein consisting of LH_N_-H_CN_/A-MH7C was shown to bind and enter rat DRG neurons, cleaved SNAP-25 and inhibited the release of CGRP. This study demonstrated the potential of using antibody fragments as targeting moieties for neurotoxins. Antibody fragments offer distinct advantages over the use of entire immunoglobulin (IgG) species, combining high specificity with small size (~25 kDa, compared to ~150 kDa), and a reduced likelihood of generating an antigenic response. This is also the first report describing the inclusion of the H_CN_ domain of the neurotoxin in a re-targeted molecule, providing an extension to the LH_N_ fragment normally utilised. The safety of LH_N_-H_CN_/A-MH7C was tested in a mouse lethality assay, where an intraperitoneal injection of 200 µg was found to be tolerated. This represents at least a 1 × 10^8^-fold decrease in lethality compared to BoNT/A [100].

A slightly different approach to altering target specificity using antibody fragments has also been described by Nugent at al. Using a recombinant protein consisting of LH_N_/A and an IgG binding moiety of staphylococcal protein A (LH_N_/A-SpA-B), this protein was then used as a binding partner to couple specific targeting moieties for the tropomyosin receptor kinase A (TrkA) receptor which is predominantly expressed in nociceptive neurons [31]. Addition of anti-tropomyosin receptor kinase A (TrkA) IgG or a fusion of Fc and a TrkA receptor ligand, nerve growth factor (NGF), (Fc-βNGF), to LH_N_/A-SpA-B was subsequently shown to target the molecule to TrkA receptors on PC-12 cells, where both coupled molecules were able to deliver LC/A resulting in SNAP-25 cleavage [31].

Moving away from neuronal targeting, a significant advance in re-targeted toxin technology was made by a TSI based on LH_N_/D containing a growth hormone releasing hormone (GHRH) ligand, Delivery of LC/D into cells was evidenced through in vitro assays by subsequent cleavage of vesicle-associated membrane protein (VAMP) proteins, which was shown to inhibit the release of growth hormone (GH) from primary rat pituicytes [39]. The effectiveness of SXN101742 was also demonstrated in vivo where the TSI, but not one with an inactive catalytic unit, produced a dose-dependent inhibition of pulsatile GH secretion in rats after receiving a single systemic injection [101].

The molecule also demonstrated long-lasting but reversible effects, key features of LH_N_-based retargeted BoNTs.

**Figure 2:**
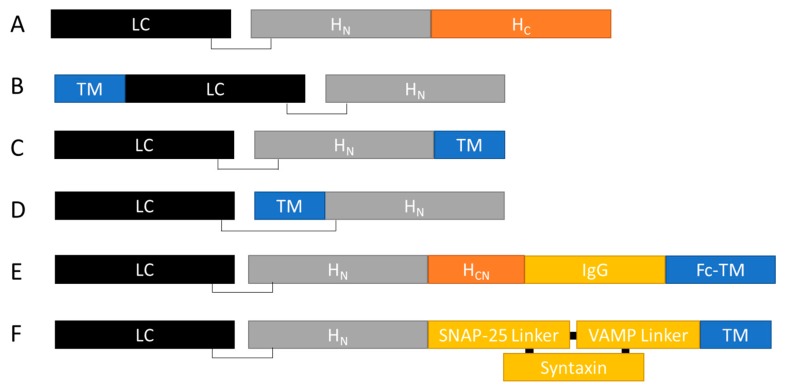
Cartoon illustration of retargeted BoNTs. Botulinum neurotoxin (**A**) can be re-targeted to different cell types by replacement of the H_C_ binding domain with an alternative targeting moiety (TM) for binding to a specific receptor. The TM (such as a peptide ligand, antibody fragment or scaffold binding protein), can be added either recombinantly, or by site-specific chemical conjugation to the N-terminal (**B**), or C-terminal (**C**) of the BoNT fragment. For TMs that require a free amino terminus for interaction with the target receptor, the TM can be centrally presented (**D**). The central TM conformation is achieved in recombinant proteins by locating the protease susceptible cleavage site directly up-steam of the TM, so that upon activation by proteolytic cleavage, the N-terminal of the TM is exposed. Other approaches for re-targeting the LH_N_ or LH_N_-H_CN_ fragment have involved the addition of an immunoglobulin (IgG) domain onto which an Fc-coupled TM can be added (**E**). A stapled peptide method, utilises an LH_N_ fragment with a SNAP-25 linker sequence which can be combined with a VAMP linker-TM fusion in the presence of a syntaxin-derived ‘stapling peptide.’ This results in an LH_N_-TM protein stapled together via a stable SNARE complex (**F**).

A completely different approach to retargeting BoNT fragments has been achieved using a stapled peptide method, which utilises the high specificity and affinity of the SNARE binding complex to form stable fusion proteins [102,103]. For a cartoon illustration of this approach and the ones discussed above, please see Figure 2. This stapling method was utilised to add a range of neuropeptides and growth factors to LH_N_/A, such as EGF, dynorphin, tumour necrosis factor (TNF), substance P, somatostatin and corticotropin releasing hormone (CRH) [104]. When added to a range of neuroblastoma and neuroendocrine cell lines, these stapled peptides displayed activities which could be rationalised by the expression patterns of the target receptors within each cell type. The molecules were also shown to target and enter different sub-populations of rat cortical neurons in contrast to BoNT/A which indiscriminately entered all neuronal cells within the culture.

The potential for retargeted BoNTs to provide novel therapeutics for a range of disorders remains high. What is abundantly clear from the combined literature on re-targeted toxins, however, is that these molecules do not share the extraordinary potency displayed by native BoNT in their activity in the target cell. TSIs and other re-targeted toxins do not deliver LC into the cytosol of their target cell as efficiently as BoNT does in neurons. This difference is not driven by any deficit in the catalytic activity of the LC, nor is it likely due to deficiencies in the affinity of target binding—indeed, where reported, binding affinities of re-targeted toxins are often higher than those estimated for BoNT binding to its receptors. Efficient internalisation of TSIs has also been demonstrated in a range of different cell types as evidenced by immunocytochemistry and high content screening methods [39,105].

It is likely that the LH_N_-ligand construct will follow the internalisation pathway of the cell surface receptor that it binds to. This has been suggested for TSIs harbouring an epidermal growth factor (EGF) ligand, which are shown to bind to cell surface EGF receptors and co-localise with EGFR in early endosomes following cellular internalisation [105]. These pathways are entirely different from the entry route of native BoNT, which enters via the synaptic vesicle recycling pathway. Such differences may account for the deficiencies in intracellular activity seen when using re-targeted toxins compared to the native neurotoxin in neurons, as the translocation of LC from these disparate internalised compartments may not be as efficient as the route of entry taken by the native toxin. One of the challenges for designing future therapeutics based on re-targeted BoNTs is to improve the efficiency with which LC is delivered following internalisation via the targeted receptor.

## 5. SNARE Cleavage Activity

**Figure 3:**
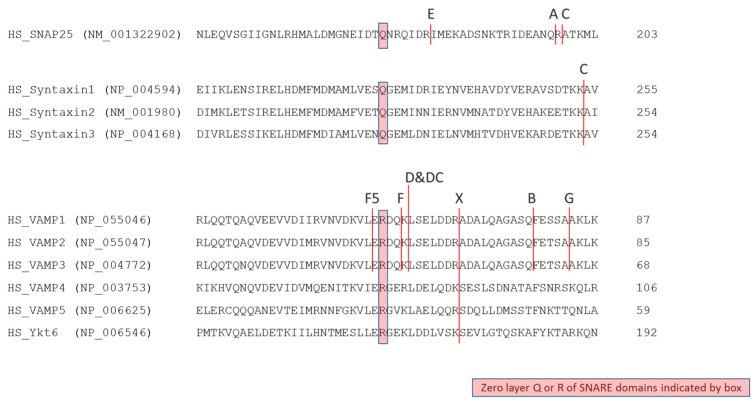
BoNT cleavage sites on SNAREs. The figure shows protein sequences from human SNARE proteins, which are targets of known naturally occurring BoNTs. BoNT cleavage sites are indicated by vertical lines. Numbers at the right of each sequence show the amino acid position of the most C-terminal shown residue within each SNARE protein. National Center for Biotechnology Information (NCBI) Reference Sequence accession numbers for each SNARE protein are in parenthesis at the left of each sequence.

As previously described, BoNTs ability to cleave members of the SNARE protein family with high selectivity is essential for their neurotoxicity. Specificity of cleavage can be limited to a single SNARE at a single site or can include multiple SNAREs and sites, see Figure 3.

**Figure 4:**
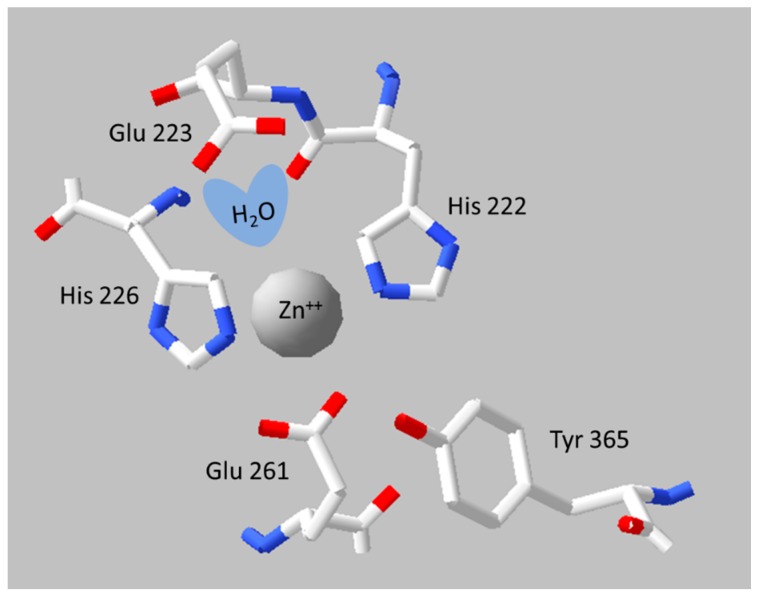
Active site of BoNT/A1. The figure shows a representation of side chains comprising the catalytic site of BoNT/A1 from crystal structure 3BTA [109]. The presumed position of a catalytically important water molecule is superimposed on the structure. The image was generated using the DeepView/Swiss-Pdb Viewer software (Version 3.7, Glaxo Smith Kline, London, UK, ©1995-2001, http://www.expasy.org/spdbv/) [110].

BoNT light chains are all members of the M27 family of neutral, zinc dependent, metallopeptidases, which share a conserved catalytic site zinc binding motif, of the form HExxH, and similar mechanisms of catalysis with the prototypical family member thermolysin [106,107]. The side chains of the HExxH motif, together with a water molecule (bound to the E of the motif) and another downstream E, coordinate a catalytic zinc ion at the active site, see Figure 4 [108].

BoNT light chains are unusual proteases because their high substrate selectivity is not determined by interactions at the catalytic site. BoNTs have substrate binding sites, located away from the active site, that specifically recruit the substrate. These interactions increase catalysis by reducing Km without changing catalytic turnover (Kcat). In contrast, active site interactions position the scissile bond for efficient catalysis (increasing turnover) but do not affect affinity for the substrate (Km) [111]. Understanding the structural determinants of selectivity and catalytic efficiency are key to engineering new BoNTs with new properties. The structures and protein chemistry of substrate binding and catalytic sites of many BoNT proteins are well understood (see for example the following references [112,113,114,115,116,117,118,119,120,121,122,123,124,125]) and can inform rational design of modified recombinant proteins.

Recent innovations in high-throughput DNA sequencing and bioinformatics are leading to the identification of increasing numbers of new BoNTs through sophisticated searches of genomic databases, rather than traditional biochemistry [42]. BoNT/X (GenBank: BAQ12790.1) is one such newly identified BoNT. It was found as an unexpressed open reading frame in the whole genome sequence of Clostridium botulinum type B strain 111 and has subsequently been produced recombinantly. Understanding BoNT/X is important from a protein engineering perspective because it cleaves an expanded set of SNARE substrates. In addition to VAMPs 1, 2, and 3, which it cleaves at position R66–A67 in VAMP2, BoNT/X also cleaves the homologous bond (relative to the SNARE zero-layer R residue [126], in VAMP4 (K87–S88), VAMP5 (R40–S41), and Ykt6 (K173–174) [30]. The X-ray crystal structure for LC/X shows a conserved fold with other BoNT LCs. Access to the catalytic site is more restricted in the LC/X structure compared to other serotypes and the regions lining the catalytic pocket are not conserved, which may underlie the unusually broad substrate selectivity [127].

Excitingly, in silico approaches are also identifying BoNT-like neurotoxins in non-Clostridial species. eBoNT/J, encoded by Enterococcus, is a BoNT-like toxin identified in silico that shows closest relationship to BoNT/X, among BoNTs, however this protein has not yet been tested biochemically and information about its SNARE cleaving activity is unavailable [128]. *Weissella oryzae*—which is a facultative anaerobic microorganism—isolated from fermenting rice, encodes a BoNT-like toxin called BoNT/Wo (NCBI Ref Seq: WP_027699549.1), which cleaves VAMP2 at W89-W90 [129]. Other newly identified non-clostridial BoNT-like toxins include *Enterococcus faecium* BoNT-like toxin (GenBank: OTO22244.1), which cleaves VAMP2 and SNAP25 [7] and *Chryseobacterium pipero* BoNT-like toxin (NCBI Ref.Seq: WP_034687872.1).

Mechanistic understanding of SNARE cleavage by the naturally occurring BoNT light chains opens opportunities for protein engineering to fine tune the activity and create new tools for biological research and medicine. One of the most obviously useful modifications is to make endopeptidase inactive versions of light chains and full-length BoNTs. This provides non-toxic proteins that can be handled safely for structural and biochemical studies that do not rely on SNARE cleavage. As BoNTs are classified, by the US Government Centres for Disease Control Department of Health and Human Services, as Category A Tier 1 select agents and subject to stringent controls by the US and most other governments, many laboratories can only work on inactivated proteins. The United States Centres for Disease Control and Prevention maintains a list of point mutations within BoNT light chains, which they recognise officially as non-toxic (https://www.selectagents.gov/exclusions-hhs-nontoxic.html#botulinum). The list includes the following mutations and supporting citations: H223A, E224A, H227A in BoNT/A [130]; E446A, H449G, Y591A in BoNT/C; H223A, E224A, H227A in BoNT/A [131,132]; E224A, Y366A in BoNT/A; R362A, Y365F in BoNT/A [133]. Several other effective inactivating mutations are also widely used in the field, examples include E224Q, H227Y in BoNT/A [132,134,135] and R372A, Y375F in BoNT/C1 [136].

The converse of inactivating mutations, are modifications designed to increase the catalytic activity of BoNT light chains. The S201P mutation in LC/B increases catalytic activity, at least 3 to 4-fold, by increasing Kcat without changing Km [137]. This was attractive because in medical use significantly higher molar doses of BoNT/B products are needed compared to BoNT/A and the possibility of creating a more potent BoNT/B suggested a potential for a new product with reduced protein load. However, the increased in vitro activity did not translate into increased potency in cell, ex-vivo, or in-vivo assays when LC/B-S201P was incorporated into full-length BoNT, which suggests that other steps in the BoNT/B mechanism of action may be limiting for the current medical uses [138].

Another highly desirable set of light chain modifications are to change the SNARE substrate selectivity, in particular to target non-neuronal SNAREs such as human SNAP23. The combination of a re-targeted BoNT with a modified light chain able to cleave specific non-neuronal SNAREs opens an enormous range of potential medical uses to treat diseases of over secretion beyond the neurology field [139]. Detailed understanding of enzyme-substrate interactions between BoNT/E and BoNT/A light chains with SNAP25 has allowed rational design of engineered BoNTs able to target SNAP23. In the case of LC/E, notable differences between SNAP25 and SNAP23 at the P2, P’2, and P’3 positions informed an engineering strategy. The P2 in SNAP25 is D (which is acidic), whereas the equivalent position in SNAP23 is K (which is basic). The S2 pocket in the LC/E active site is basic, containing K224, and makes a predicted salt bridge with SNAP25 P2 D179 but is expected to repel the SNAP23 P2 position residue K185. A modified LC/E, with a K224D mutation, was engineered to correct the charge clash with SNAP23 and this engineered protein can cleave human SNAP23 [140]. The challenge for BoNT/A was greater because SNAP23 resistance to this light chain was not due to catalytic site interactions but rather to inefficient substrate binding at multiple exosite interactions (leading to low Km). Based on the SNAP25-LC/A crystal structure, dynamic structural modelling, and systematic screening of mutations in predicted exosite interaction pockets, engineered LC/A proteins have been created with enhanced human SNAP23 binding and cleavage activity [113]. 

BoNT/C1 is cytotoxic in neurons, but not non-neuronal cells, due to inactivating both SNAP25 and syntaxin 1 [141,142,143]. A large methodical, directed mutagenesis approach that tested more than 150 modified LC/C1 proteins designed based on modelling LC/C1 against the SNAP25-LC/A structure [108], identified two triple mutations in LC/C1 that retained high levels of syntaxin 1 cleavage but did not cleave SNAP25. These proteins were named BoNT/C1α-3W (L200W, M221W, I226W), and BoNT/C1α-51 (residues T51, N52, P53 mutated). The BoNT/C1α-3W residues map to the LC/C1 S’1 catalytic site pocket, whereas the BoNT/C1α-51 residues are in a previously relatively unexplored region [136].

Understanding the SNARE cleavage activity of BoNTs opens the potential to increase the therapeutic scope of medical uses for newly designed BoNTs into non-neuronal disease areas. It also offers the opportunity to generate safer and more precise biological tools to dissect the functions of SNARE proteins. This may in turn lead to new insights into cellular physiology and offer better understanding of diseases of secretion.

## 6. Conclusions

Engineering of BoNTs has been made possible by recombinant technologies. Firstly, wild type expression of full length BoNTs and their individual domains, together with point mutations and domain swaps, has allowed the advancement of BoNT used as standards and as future therapeutics. Secondly, the ability to choose different hosts to produce BoNTs will likely revolutionise future manufacturing of BoNTs for clinical use. Finally, the improvement and expansion of both cell targeting and substrate cleavage activity of BoNTs will open opportunities up for therapies able to affect tissues beyond the neuronal system.
